# Photoprotective Effects of *Dendrobium nobile* Lindl. Polysaccharides against UVB-Induced Oxidative Stress and Apoptosis in HaCaT Cells

**DOI:** 10.3390/ijms24076120

**Published:** 2023-03-24

**Authors:** Yunluan Long, Wuji Wang, Yanyan Zhang, Fanpan Du, Shiqian Zhang, Zheng Li, Jiang Deng, Jingjie Li

**Affiliations:** 1 Key Laboratory of Basic Pharmacology of Ministry of Education and Joint International Research Laboratory of Ethnomedicine of Ministry of Education, Zunyi Medical University, Zunyi 563006, China; 2 Key Laboratory of Basic Pharmacology of Guizhou Province, Zunyi Medical University, Zunyi 563006, China; 3 Department of Pharmacology, School of Pharmacy, Zunyi Medical University, Zunyi 563006, China

**Keywords:** ultraviolet B (UVB), HaCaT keratinocytes, *Dendrobium nobile* Lindl polysaccharides, antioxidant, anti-apoptosis, MAPK

## Abstract

Acute ultraviolet (UV)-B radiation is the major external factor causing photodamage. In this study, we aimed to determine the effects of *Dendrobium nobile* Lindl. polysaccharides (DNPs) on photodamage in HaCaT keratinocytes after UVB irradiation and the underlying mechanisms. We found that DNPs significantly attenuated the decline in the viability and proliferation of HaCaT cells after UVB irradiation. Moreover, DNPs scavenged reactive oxygen species (ROS), improved the activities of endogenous antioxidant enzymes, including superoxide dismutase, catalase, and glutathione peroxidase, and reduced the levels of malondialdehyde, while partially attenuating cell cycle arrest, suggesting their antioxidant and anti-apoptotic properties. The mitogen-activated protein kinase (*MAPK*) pathway was found to be important for the attenuation of UVB-induced photodamage in the HaCaT cells. Furthermore, DNPs exerted cytoprotective effects by downregulating UVB-induced ROS-mediated phosphorylation of MAPKs, including p38, c-Jun N-terminal kinase, and extracellular signal-regulated kinase, and by inhibiting p53 expression as well as the apoptotic cascade response. Therefore, DNPs ameliorated UVB-induced oxidative damage and apoptosis in HaCaT cells via the regulation of *MAPKs*. Our findings thus highlight the *Dendrobium nobile* Lindl polysaccharides as promising therapeutic candidates for UVB-induced photodamage.

## 1. Introduction

Ultraviolet (UV) radiation is the main extrinsic factor responsible for sunburn, erythema, photodamage, and skin cancer [1]. Based on its wavelength, UV radiation has been classified into three types: UVA (315–400 nm), UVB (280–315 nm), and UVC (100–280 nm) [2]. UVC radiation cannot reach the ground as it is absorbed by the ozone layer, and UVB radiation possesses more energy than UVA radiation [3] and is the primary cause of skin photodamage [4].

Excessive UVB radiation causes direct damage to DNA in the skin and produces large amounts of reactive oxygen species (ROS), resulting in the activation of cellular signaling pathways associated with cell viability and death [5]. ROS play a central role in the development of oxidative stress. Skin contains an effective antioxidant defense system with various enzymes, such as superoxide dismutase (SOD), catalase (CAT), and glutathione peroxidase (GSH-Px), that can scavenge excessive ROS and maintain the balance of pro-oxidants and antioxidants in the body to prevent cell damage and subsequent apoptosis caused by the imbalance of intracellular redox in the skin [6]. This process helps in maintaining homeostasis in the organism. Apoptosis caused by UVB radiation is predominantly mediated by the intrinsic pathway in keratinocytes [7].

Mitogen-activated protein kinases (MAPKs) are an intracellular superfamily of serine/threonine protein kinases that are ubiquitous in cells of many organisms, including yeast and mammals, and are essential for the transduction of signals from cytoplasm to the nucleus [8]. ROS can activate MAPKs, including extracellular signal-regulated kinase (ERK)-1/2, c-Jun N-terminal kinase (JNK), and p38 kinase (p38) [9]. In addition to its essential function in the mediation of various cellular responses, including stress and inflammation, MAPKs are also closely associated with cell growth, development, and differentiation and can effectively regulate and inhibit the critical signaling pathways involved in the regulation of apoptosis [10], whereas p38 and JNK are also associated with the intrinsic pathway of apoptosis. The *p53* is a key tumor suppressor gene [11,12] that plays a central role in signal transduction pathways such as apoptosis. It can participate in key cellular processes such DNA damage repair, cell cycle regulation, and apoptosis via activating the transcriptional expression of its downstream gene [13]. P53 can be activated by p38 [14], so we hypothesize that apoptosis is transduced via the MAPKs/p53 signaling pathway.

*Dendrobium nobile* Lindl. (DNL), a valuable Chinese herb, which belongs to the genus *Dendrobium* in the orchid family of the Order Asparagales. It is classified as a superior product in “Shen Nong’s Herbal Classic” because of its excellent tonic properties in alleviating paralysis, lowering Qi, and tonifying the weakness of the internal organs. Previous studies on DNL have mainly focused on its small molecule components, such as phenols, alkaloids, and sesquiterpenes [15,16,17,18], while only a few studies have investigated its bioactive macromolecules. DNL polysaccharides (DNPs) are among the main active components of DNL, exerting antioxidant [19], anti-inflammatory [17], and anti-apoptotic effects [20,21]. Li [22] reported that DNPs ameliorate UVA-induced photoaging of fibroblasts. We also previously reported that DNPs significantly alleviate photodamage caused by UVB radiation in Kunming mice [23]. However, whether DNPs exert protective effects on human keratinocyte (HaCaT) cells remains unclear. Therefore, in this study, we determined the protective effects of DNPs against UVB-induced acute photodamage in HaCaT cells and elucidated the underlying mechanism.

Herein, we report on the changes in viability, proliferation, apoptosis, cell cycle, oxidative stress, and MAPK pathway-related indicators in the HaCaT cells.

## 2. Results

### 2.1. DNPs Attenuate UVB-Induced Damage in HaCaT Cells

**Figure 1:**
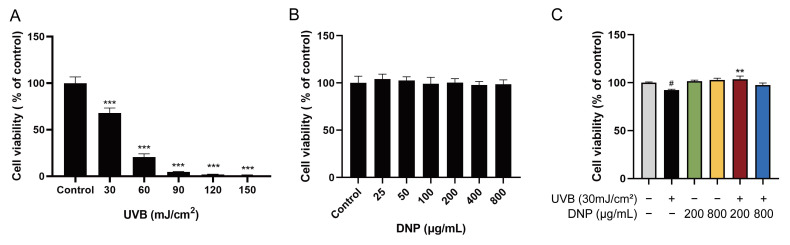
Protective effects of *Dendrobium nobile* Lindl. polysaccharides (DNPs) on the viability of ultraviolet (UV)-B-irradiated HaCaT cells. (**A**) Effects of various UVB radiation doses on the viability in HaCaT cells for 24 h. (**B**) Cytotoxicity of DNPs on HaCaT cells. (**C**) Effects of DNPs on UVB-irradiated HaCaT cells. ^#^ *p* < 0.05 and *** *p* < 0.001 vs. control group; ** *p* < 0.01 vs. UVB group.

We determined the effects of different doses of UVB irradiation on the viability of HaCaT cells using a cell counting kit (CCK)-8 assay. As the UVB radiation dose increased to 30–150 mJ/cm^2^, cell viability decreased (Figure 1A). Significant cytotoxicity was observed after 24 h of UVB exposure with more than 30 mJ/cm^2^. Therefore, we selected 30 mJ/cm^2^ of UVB radiation for subsequent experiments. As shown in Figure 1B, no cytotoxicity was observed after pre-treatment of HaCaT cells with DNP below 800 μg/mL for 24 h. We further determined whether DNPs exert any ameliorative effects on UVB-irradiated HaCaT cells by assessing their cell viability. We observed a significant increase in the viability of HaCaT cells incubated with 200 μg/mL DNP prior to UVB irradiation (Figure 1C). These findings indicate that DNPs played a key role in improving the viability of HaCaT cells after UVB irradiation.

### 2.2. DNPs Scavenge Intracellular ROS

**Figure 2:**
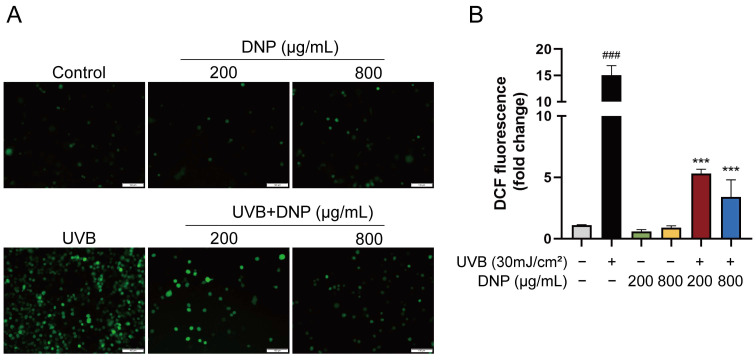
DNPs inhibit UVB-induced intracellular ROS generation. (**A**) Images of DCFH-DA fluorescence observed via microscopy. Scale bar = 100 μm. (**B**) Quantification of fluorescence intensity. ^###^ *p* < 0.001 vs. control group; *** *p* < 0.001 vs. UVB group.

Levels of ROS were significantly increased in UVB-irradiated cells by 13.6-fold compared to levels in the control group, whereas pre-treatment with DNPs reversed this effect. Compared with the UVB group, pre-treatment with 200 and 800 μg/mL of DNP prior to UVB irradiation reduced the intracellular ROS levels by 35.2% and 22.6%, respectively. Fluorescence microscopy revealed that DNP pre-treatment notably decreased the green fluorescence intensity of 2′,7′-dichlorodihydrofluorescein diacetate (DCFH-DA; Figure 2). These findings indicated that DNPs scavenged UVB-induced ROS.

### 2.3. DNPs Alleviate UVB-Induced Oxidative Stress Damage

SOD, CAT, and GSH-Px possess potent free radical-scavenging abilities and are critiIcal enzymes in defense against UVB-induced oxidative stress. MDA, which is the main sub-metabolite of lipid peroxidation, is a biomarker for assessing oxidative damage in cell membranes, as it reduces the fluidity of cell membranes, impairs material exchange, and ultimately leads to cell rupture and death. Therefore, levels of SOD, CAT, GSH-Px, and MDA were determined to assess the extent of oxidative damage. To determine whether DNP pre-treatment attenuates UVB-induced oxidative stress indicators, the enzymatic activities of SOD, CAT, and GSH-Px, and the levels of MDA were measured in all groups of HaCaT cells using specific kits.

**Figure 3:**
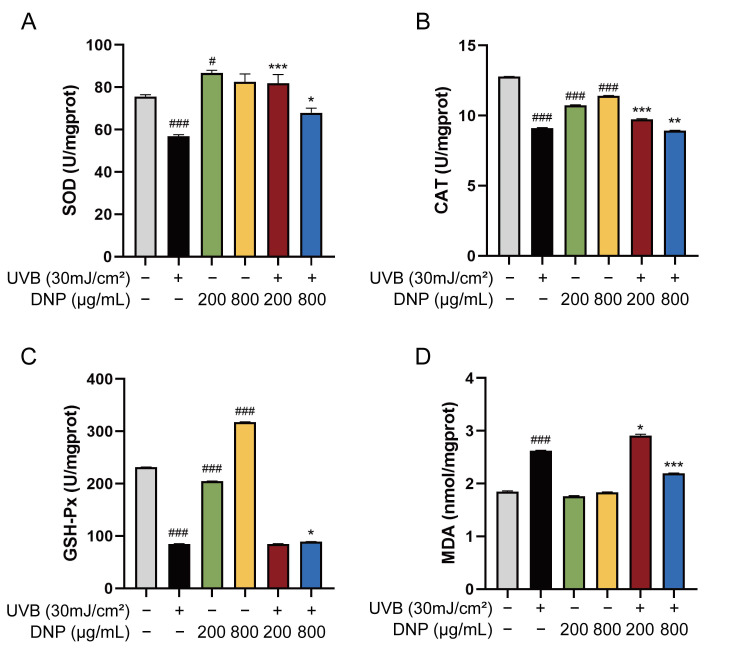
DNPs alleviate UVB-induced oxidative damage in HaCaT cells. Activities of (**A**) SOD, (**B**) CAT, and (**C**) GSH-Px. (**D**) Malondialdehyde (MDA) levels. ^#^ *p* < 0.05, and ^###^ *p* < 0.001 vs. control group; * *p* < 0.05, ** *p* < 0.01 and *** *p* < 0.001 vs. UVB group.

Activities of endogenous antioxidant enzymes in the UVB group decreased to 56.86 U/mg protein for SOD, 9.11 U/mg protein for CAT, and 84.69 U/mg protein for GSH-Px vs. control group (*p* < 0.001; Figure 3). In contrast to its effects on antioxidant enzyme activities, UVB increased the MDA levels 1.42-fold compared to those in the control group. However, on incubation with 200 μg/mL DNP prior to UVB irradiation, the reduction in SOD and CAT enzyme activities by UVB irradiation was significantly attenuated in the cells. As compared to the UVB group, the enzymatic activity of GSH-Px increased slightly after pretreatment with 800 μg/mL DNP for 24 h followed by UVB irradiation, with an activity of 89.03 U/mg protein (*p* < 0.05). Similarly, the UVB-induced increase in MDA levels was reversed by pre-treatment with DNP at 800 μg/mL (*p* < 0.001). Furthermore, incubation with 200 μg/mL DNP without UVB irradiation significantly enhanced SOD activities compared with the control group, while for GSH-Px activity, 800 μg/mL DNP was required in HaCaT cells. These findings indicate that DNPs alleviate UVB-induced oxidative stress by improving the activities of endogenous antioxidant enzymes and decreasing the MDA levels in HaCaT cells.

### 2.4. DNPs Rescue UVB-Induced Cell Cycle Arrest

**Figure 4:**
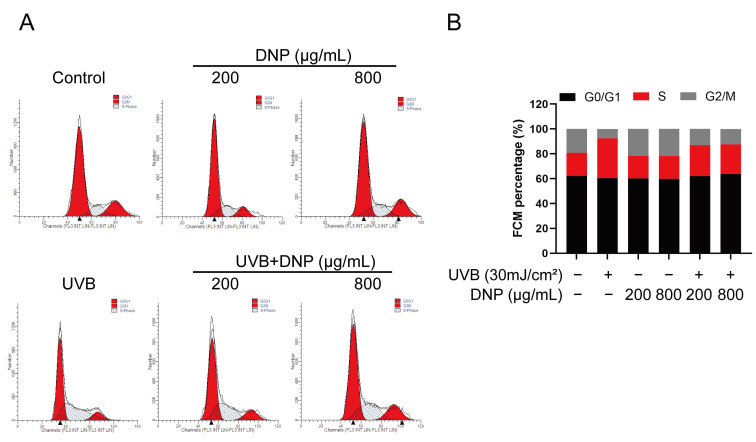
DNP pre-treatment rescues UVB-induced cell cycle arrest. (**A**) Cell cycle analysis of HaCaT cells subjected to different treatments using flow cytometry. (**B**) Statistical analysis of cells in different phases of the cell cycle.

Flow cytometry revealed that UVB irradiation arrested the cell cycle in HaCaT cells (Figure 4). We assessed the cell cycle distribution of UVB-irradiated HaCaT cells after DNP pre-treatment. G0/G1 phase was the predominant phase of the cell cycle, with a 72.3% increase in cells in the S phase vs. the control group, and a decline in cells in the G2/M phase, indicating that the cell cycle was arrested in the S phase. Pre-treatment with DNP considerably alleviated UVB-induced cell cycle arrest by decreasing the number of cells in the S phase (22.3% for 200 μg/mL DNP and 25.6% for 800 μg/mL DNP) and increasing the number of cells in the G2/M phase (72.8% for 200 μg/mL DNP and 64.2% for 800 μg/mL DNP) compared with those in the UVB group (Figure 4).

### 2.5. DNPs Inhibit UVB-Induced Activation of MAPKs

**Figure 5:**
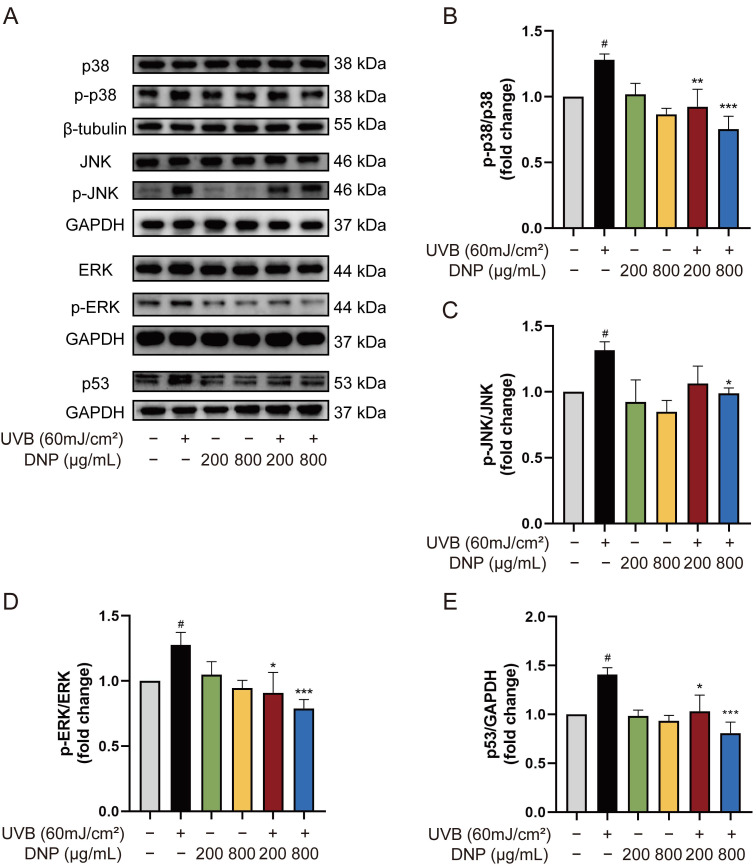
DNPs exert protective effects by regulating MAPK and p53 pathways. (**A**) Protein expression levels of MAPKs and p53. Statistical analysis of the phosphorylation levels of p38 (**B**), JNK (**C**), ERK (**D**), and p53 (**E**). ^#^ *p* < 0.05 vs. control group; * *p* < 0.05, ** *p* < 0.01, and *** *p* < 0.001 vs. UVB group.

*MAPK* and *p53* signaling pathways are essential transduction pathways activated by UVB irradiation and contribute to skin photodamage. Therefore, we explored the effects of DNP on UVB-induced abnormal phosphorylation of the *MAPK* and *p53* pathways in HaCaT cells in this study (Figure 5). Compared with the control group, UVB radiation significantly increased the expression of p53 and phosphorylation of p38, JNK, and ERK1/2 (*p* < 0.05), whereas pre-treatment with DNP reversed these effects. DNPs significantly inhibited p53 expression with increasing doses and decreased the p53/GAPDH ratio to 73.4% and 57.3% of those the UVB group, respectively. Nevertheless, pre-treatment of cells with 800 μg/mL DNP significantly decreased the phosphorylation of p38, JNK, and ERK by 58.8%, 75%, and 61.9% of the original levels, respectively, compared to those in the UVB group. Notably, pre-treatment with 200 μg/mL DNP prior to UVB irradiation significantly decreased the phosphorylation levels of ERK1/2 and p38, but not JNK.

### 2.6. DNPs Inhibit UVB-Induced Apoptosis of HaCaT Cells

**Figure 6:**
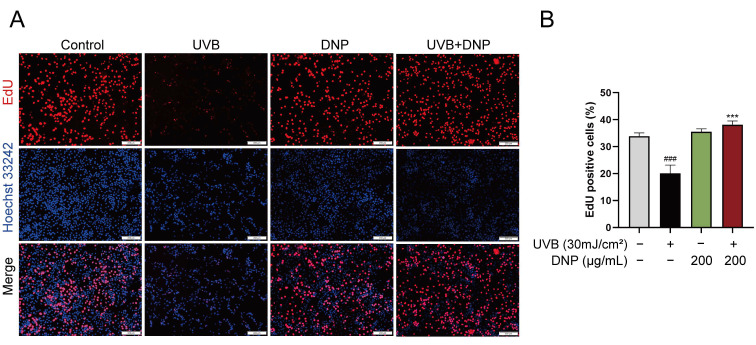
DNPs promote HaCaT cell proliferation. (**A**) Representative images of EdU staining. Proliferating cells are stained red, and cell nuclei are stained blue. Scale bar = 200 μm. (**B**) Statistical analysis of EdU-positive cells. ^###^ *p* < 0.001 vs. control group; *** *p* < 0.001 vs. UVB group.

We performed a 5-ethynyl-2′-deoxyuridine (EdU) assay to determine the effects of DNP on DNA synthesis and cell proliferation after UVB irradiation. EdU staining and positivity in HaCaT cells with different treatments are shown in Figure 6. Compared with the control group, which showed luminescent and specific nuclear staining, the UVB group exhibited a weak signal. UVB irradiation significantly decreased the EdU-positive nuclei (approximately 50%), indicating that UVB irradiation blocked cell proliferation and did not stimulate DNA synthesis. However, these effects were reversed by DNP (*p* < 0.001). These findings indicated that DNP pre-treatment inhibited UVB-induced impairment of DNA synthesis and cell proliferation.

**Figure 7:**
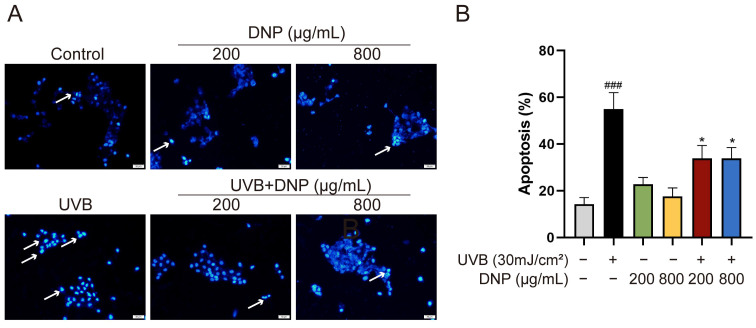
DNPs inhibit UVB-induced apoptosis of cells. (**A**) Representative images of Hoechst 33258 staining. The blue region indicates cell nuclei, and white arrows indicate the apoptotic cells with distinct features, such as intense fluorescence and presence of apoptotic bodies. Scale bar = 50 μm. (**B**) Quantification of apoptosis. ^###^ *p* < 0.001 vs. control group; * *p* < 0.05 vs. UVB group.

UVB-induced oxidative stress has been reported to cause cell death via apoptosis. To determine the effect of DNP pre-treatment on UVB-induced cell apoptosis, we stained the cell nuclei with Hoechst 33258 fluorescent dye and assessed the formation of apoptotic bodies, typical morphological markers of apoptosis. Hoechst 33258 specifically binds to DNA and can identify apoptotic nuclei with DNA breaks and condensed chromatin. Compared with the controls, condensed chromatin, shrunk cells, and apoptotic vesicles were observed in UVB-irradiated HaCaT cells (Figure 7). However, pre-treatment with DNP before UVB irradiation significantly reduced the rate of apoptosis (*p* < 0.05).

Apoptosis is an important pathological process for skin photodamage. Therefore, we used an immunofluorescence assay to determine whether DNPs can protect against UVB-mediated apoptosis via the suppression of key molecules, such as caspases, for the regulation of apoptosis. We determined the expression levels of cleaved caspase-9, which is involved in the endogenous apoptotic pathway. Additionally, we determined the levels of cleaved caspase-3, which plays a central role in apoptosis.

**Figure 8:**
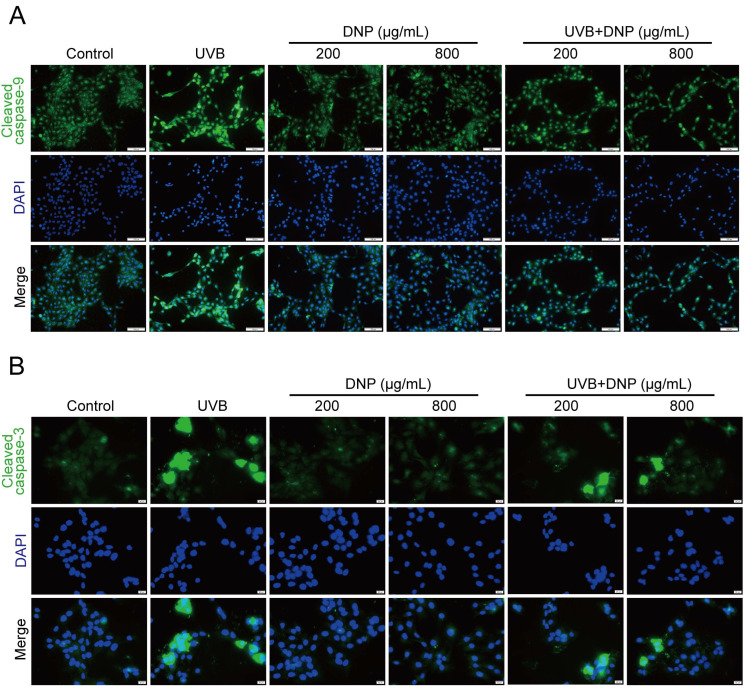

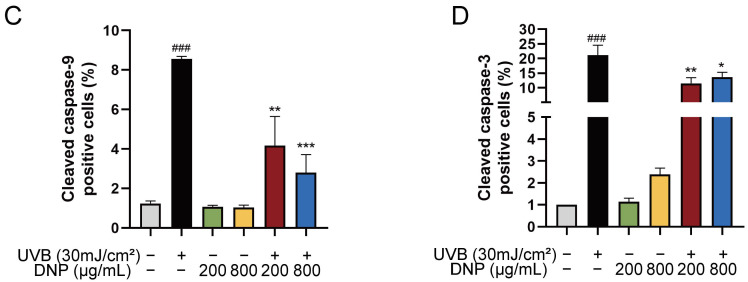
DNPs inhibit UVB-induced apoptosis-related proteins expression in HaCaT cells. (**A**) Representative fluorescence images showing cleaved caspase-9 staining and (**B**) cleaved caspase-3 staining. Scale bar = 100 μm (**A**)/20 μm (**B**); Cleaved caspase-9 and cleaved caspase-3 positive cells were counted (**C**,**D**). ^###^ *p* < 0.001 vs. control; * *p* < 0.05, ** *p* < 0.01, and *** *p* < 0.001 vs. UVB.

Compared with the controls, significant upregulation of cleaved caspase-9 and cleaved caspase-3 expression levels was observed after 6 h of UVB irradiation in HaCaT cells (Figure 8). Notably, all concentrations of DNP inhibited the UVB-induced upregulation of cleaved caspase-3 and cleaved caspase-9 levels. Furthermore, 200 μg/mL DNP downregulated the expression levels of cleaved caspase-9 and cleaved caspase-3 to 48.7% and 54.4%, respectively, whereas 800 μg/mL DNP decreased their expression levels to 32.7% and 64.5%, respectively. These findings suggested that DNPs protected against UVB-induced apoptosis via the inhibition of caspase-9 and caspase-3 activation, thereby only partially activating the pro-apoptotic signaling cascade.

## 3. Discussion

UVB, the main external factor causing skin damage, has a poor penetration capacity and can only reach the epidermal layer of the skin [24]. Epidermal cells are mainly composed of keratinocytes, which are responsible for the absorption of most UVB radiation, resulting in dryness, inflammation, and apoptosis [25]. HaCaT is a keratinocyte line that is widely used to investigate the cellular responses to UVB radiation as well as the underlying mechanisms [26,27]. UVB irradiation causes many diseases including sunburn, photodamage, and cancer, by altering the signal transduction associated with oxidative stress, cell cycle arrest, and cell apoptosis. Therefore, attenuation of oxidative stress and apoptosis caused by UVB exposure is a prospective strategy to protect against cell damage. We evaluated the photoprotective effects of DNP on UVB-induced HaCaT cells. We found that DNPs attenuated oxidative stress and apoptosis induced by UVB radiation via the *MAPK* signaling pathway.

UVB induces oxidative stress and causes damage to cells via ROS production [28,29,30]. Antioxidant enzymes in the human skin are critical for balancing ROS levels in cells and protecting against oxidative stress caused by UVB irradiation to maintain homeostasis [31]. Overexposure to UVB radiation beyond the limits of cutaneous antioxidant capacity leads to excess ROS production with oxidative capacities greater than the antioxidative capacities of endogenous enzymes, such as SOD, CAT, and GSH-Px [32], as well as biomolecules, such as proteins, lipid membranes, and mitochondria, in cells [33,34,35]; this leads to extensive cellular oxidative damage and apoptosis. ROS levels increase rapidly after UVB irradiation for a short period of time, decrease substantially within 1 h, and become stable over time, reaching the same values as in the control group 90 min after UVB exposure [36]. Consistent with a previous study [37], we found that ROS accumulated rapidly in HaCaT cells after UVB irradiation for 15 min, while DNPs significantly inhibited ROS production. In addition, ROS have a strong oxidative capacity and can induce lipid peroxidation of polyunsaturated fatty acids in biological membranes [38,39] and produce MDA, the end product of lipid peroxidation; therefore, MDA is also one of the most commonly used indicators of lipid peroxidation [40]. MDA can undermine the cell membrane, which is critical to the cell as it acts as a barrier against the extracellular environment and can lead to cell death. In line with the findings of Tebbe [41], UVB at an intensity of 30 mJ/cm^2^ produced dramatic increases in MDA levels and suppressed endogenous antioxidant enzyme activity. Keratinocytes are more sensitive to oxidative stress than fibroblasts because of their low antioxidant enzyme activity [42]. Our study showed that pre-treatment with DNP in HaCaT cells restored the enzymatic activities of SOD, CAT, and GSH-Px and significantly decreased the level of MDA. This suggested that DNPs could counteract oxidative stress and inhibit the development of lipid peroxidation induced by UVB radiation. We speculated that DNPs may directly reduce the production of ROS and indirectly restore the activities of antioxidant enzymes. Furthermore, DNPs reduced the UVB-induced increase in MDA content not only because of their direct effect on MDA, but also possibly due to their inhibition of ROS production and thus reduced MDA content. These results suggested that DNPs could protect against UVB-induced oxidative stress and lipid peroxidation, thereby alleviating oxidative damage.

UVB radiation stimulates the MAPK signaling pathway in a direct or indirect manner via an ROS-mediated pathway [9,43]. MAPKs continuously phosphorylate a cascade of reactions that engage in various physiological events, including cell growth, development and apoptosis [44]. It has been reported that acute UV radiation exposure can activate the p38, JNK, and ERK signaling pathways within 1 h [45]. Indeed, activation of ERK signaling is essential for regulating cell viability and proliferation [46]. Our results showed that DNP pre-treatment significantly downregulated the hyperphosphorylation of p38, JNK, and ERK in HaCaT cells after 1 h of UVB irradiation and also partially inhibited the UVB-induced decrease in cell viability and the rate of EdU-positive cells associated with DNA synthesis. These results suggest that DNPs may inhibit the reduction in cell viability and proliferation by downregulating the phosphorylation of ERK induced by UVB irradiation. UVB can damage DNA directly or indirectly, and most cells exposed to DNA damage have elevated levels of p53, which promotes the transcription and expression of the cell cycle protein-dependent kinase inhibitor p21, thereby regulating cell cycle progression in both G1 and S phases. Activation of the ERK signaling pathway likewise induces the expression of cell cycle inhibitory proteins, leading to cell cycle arrest [47,48,49]. Consistently, we showed that DNPs ameliorated UVB-induced S-phase cell cycle arrest via the downregulation of UVB-induced phosphorylation of the *ERK* pathway and p53 protein expression.

UVB radiation can trigger apoptosis through the activation of intrinsic pathways associated with DNA damage, extrinsic pathways [50] or ROS production [7,51,52]. However, some evidence suggests that UVB-induced apoptosis in keratinocytes primarily activates the intrinsic apoptotic pathway, and that activation of this pathway correlates with the activation of Pro caspase-9 [7]. In general, activation of the *p38* and *JNK* signaling pathways is responsible for the initiation of UVB-induced apoptosis. Stimulation of p38 and JNK causes the release of cytochrome C, which triggers caspase-9 and caspase-3, and leads to intrinsic apoptosis. Cleaved caspase-9 initiates the caspase cascade reaction, where caspase-9 enzymatically cleaves caspase-3 precursors and then releases the C-terminal small peptide fragment, thereby triggering caspase-3 and finally resulting in apoptosis [53]; cleaved caspase-3 could activate caspase-9 zymogen, thus forming a positive feedback pathway. Cleaved caspase-3 accelerates the separation of damaged cells from the surrounding tissues and ultimately leads to the formation of apoptotic vesicles. Therefore, coagulation of nuclei and formation of apoptotic vesicles are also characteristics of apoptosis. In our study, the results of Hoechst 33258 staining were consistent with those in the literature, with the aforementioned nuclear sequestration and apoptotic vesicles in HaCaT cells after 6 h of UVB irradiation, as well as a significant increase in the expression of cleaved caspase-9 and cleaved caspase-3. In contrast, DNP pre-treatment significantly downregulated the number of apoptotic vesicles and the expression of the apoptosis marker proteins, cleaved caspase-9 and cleaved caspase-3. The *p53* gene is closely associated with apoptosis, and cells with high p53 expression undergo apoptosis [54,55]. In addition, p38 increases c-Myc levels via upregulation of p53 expression, and elevated c-Myc levels further enhance the activity of cleaved caspase-3 and promote apoptosis [56]. ERK activation is associated with cell apoptosis [57,58,59].

**Figure 9:**
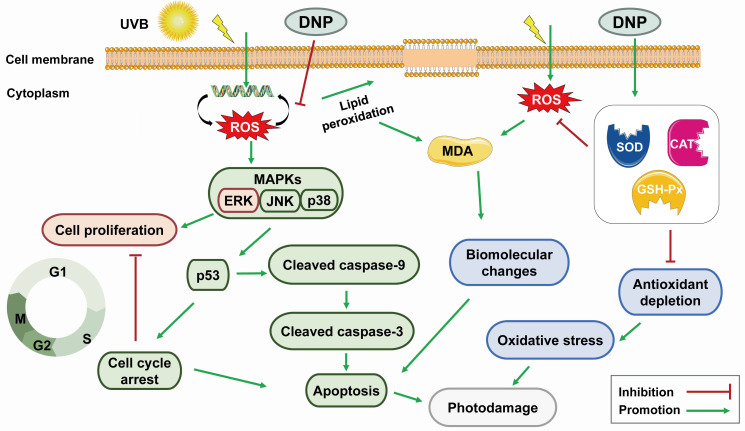
Potential mechanism of DNP in ameliorating the UVB-induced photodamage of HaCaT cells.

Overall, our results revealed that DNPs decreased the production of apoptotic vesicles, upregulated the expression levels of cleaved caspase-9 and cleaved caspase-3, and downregulated the *MAPK* pathway and *p53* expression levels, indicating the protective effects of DNP against UVB-induced photodamage in HaCaT cells (Figure 9).

## 4. Materials and Methods

### 4.1. Materials

Dulbecco’s modified Eagle’s medium (DMEM) and trypsin-EDTA were purchased from Gibco (Shanghai, China). Fetal bovine serum (FBS) was supplied by Adamas Life (C8010; Shanghai, China). Penicillin/streptomycin and ROS assay kits were obtained from Solarbio Co., Ltd. (Beijing, China). Hoechst 33258 staining solution, Cell Cycle and Apoptosis Analysis Kit, and BeyoClick EdU Cell Proliferation Kit with Alexa Fluor 555 antibody were obtained from Beyotime Biotechnology (Shanghai, China). SOD (A001-3-2), CAT (A007-1-1), GSH-Px (A005-1-2), and MDA (A003-1-2) assay kits were obtained from the Jiancheng Biotechnology Institute (Nanjing, China). DNPs were obtained from Shanghai Winherb Medical Science Co. Ltd.

### 4.2. Cell Culture 

HaCaT cells, provided by Shanghai Gaining biological technology Co., Ltd.(Shanghai, China), were cultured in DMEM supplemented with 10% FBS and 1% penicillin/streptomycin at 37 °C in a humidified 5% CO_2_ incubator. HaCaT cells were collected when they reached 70–80% confluency.

### 4.3. UVB Irradiation

HaCaT cells were incubated in 60-mm cell culture dishes with the complete medium until they reached 80% confluency. Cells were incubated with DNP or vehicle (DMEM containing 1% antibiotics) for 24 h before UVB irradiation. Cells were then washed with phosphate-buffered saline (PBS) and mixed with 2 mL of PBS to completely cover the bottom of the cell culture dishes to prevent the drying of the cell surface. The lid of the Petri dish was opened, and placed at the center of the Bio-Link Crosslinker (Vilber Lourmat, Cedex, France), which emitted 312 nm radiation. All cells were irradiated with the corresponding UVB dose. Both control and DNP-only groups were excluded from UVB radiation. Following UVB irradiation, the cells were cultivated in a serum-free medium for subsequent experiments.

### 4.4. Cell Viability Assay

The viability of HaCaT cells subjected to different treatments was assessed using the CCK-8 (Dojindo Laboratories, Kumamoto, Japan). To assess the cytotoxicity of DNP, cells were incubated with various concentrations of DNP (25, 50, 100, 200, 400, and 800 μg/mL) in a medium containing 1% antibiotics for 24 h. Cells were cultivated in a serum-free medium for 24 h after irradiation with different doses of UVB. To assess the effects of DNP on UVB-irradiated cells, the cells were pre-treated with DNP for 24 h followed by UVB irradiation at an appropriate dose. Then, cells were incubated in 100 µL of DMEM supplemented with 10 μL CCK-8 solution at 37 °C for approximately 1.5 h and the optical density was mesaured at 450 nm using a microplate spectrophotometer (Thermo Scientific, Waltham, MA, USA).

### 4.5. Determination of Intracellular ROS Levels

ROS levels were determined using the fluorescent probe, DCFH-DA. Cells were incubated with DNP for 24 h, followed by exposure to UVB radiation. Cells were again incubated with 10 μM DCFH-DA at 37 °C for 30 min in the dark, washed with PBS twice, and observed using an inverted fluorescent microscope (IX73+DP73; Olympus, Tokyo, Japan).

### 4.6. Measurement of Oxidative Stress-Related Marker

HaCaT cells (at least 10^6^ cells) were subjected to different treatments. Cells were lysed using the radioimmunoprecipitation assay (RIPA) buffer (Solarbio); supernatant was centrifuged and collected for the protein concentration assay, and oxidative stress-related biomarker levels were measured using kits. 

For MDA assay, 10 nmol/L standard, anhydrous ethanol, cell supernatant, and reagent were added to the cells and mixed, followed by the addition of the reagent application solution and 50% glacial acetic acid. Cells were centrifuged, vortexed, heated in a 95 °C water bath for 80 min, and cooled down under running water. Centrifugation with 3000× *g* was performed to collect the supernatant for the measurement of absorbance at 523 nm.

For the determination of SOD activity, all reagents and samples were added to the 96-well plate and cells were incubated at 37 °C for 20 min, according to the manufacturer’s instructions. Absorbance was measured at 450 nm using a microplate spectrophotometer.

For the measurement of CAT activity, the reaction was set up with control and sample tubes. Cell supernatant and reagents Ⅰ and Ⅱ pre-warmed at 37 °C were added and reacted in water bath at 37 °C for 1 min. Subsequently, reagent III, reagent IV, and the cell supernatant were added sequentially and mixed. A UV–Vis spectrophotometer (HITACHI, Tokyo, Japan) was used to determine the absorbance of the samples at 405 nm.

For the determination of GSH-Px activity, enzymatic reactions were first performed by setting up non-enzymatic and enzymatic tubes. After adding 1 mmol/L GSH or cell supernatant and reagent Ⅰ solution pre-warmed at 37 °C for 5 min, the reaction was carried out in a water bath at 37 °C for 5 min. After subsequent incubation of reagent II solution and cell supernatant and subsequent centrifugation, 1 mL of supernatant was used for the color development reaction. The color development reaction was set up using blank and standard tubes, the specified reagents were added, and mixed. Absorbance of the samples was determined at 412 nm after incubation at room temperature for 15 min.

### 4.7. Cell Cycle Analysis

Cells were treated according to their group, trypsinised with trypsin-EDTA, and a minimum of 10^6^ cells was collected for subsequent experiments. Cells were fixed with 70% pre-chilled ethanol at 4 °C for 30 min, centrifuged with 1000× *g* for 10 min to remove the supernatant, resuspended in pre-chilled PBS, and centrifuged again. Subsequently, the cells were resuspended in a propidium iodide staining solution containing RNase A, incubated at 37 °C for 30 min in the dark, and filtered through a 70-mesh sieve into a flow tube. Flow cytometry (Navios; Beckman Coulter, Brea, CA, USA) was used to assess the DNA content, and cell cycle distribution was analyzed using Moditfit LT V3.2 software.

### 4.8. Hoechst 33258 Staining

After reaching 80% confluency, HaCaT cells were incubated with DNP or vehicle for 24 h and exposed or not exposed to UVB. The supernatant was discarded. The cells were washed in PBS, fixed with 4% paraformaldehyde for 15 min, and incubated with Hoechst 33258 solution for 10 min in the dark. Changes in the nuclear morphology of HaCaT cells were observed using an inverted fluorescence microscope (IX73+DP73; Olympus, Tokyo, Japan).

### 4.9. EdU Staining

EdU staining was performed to assess the proliferation of HaCaT cells under different conditions. After treatment according to their group, cells were incubated with pre-warmed 2× EdU working solution (20 μM) at 37 °C for 2 h, fixed with 4% paraformaldehyde (Solarbio) for 15 min, and permeabilized with 0.3% Triton X-100 (Sigma-Aldrich, Shanghai, China) for 10 min at room temperature. Then, the supernatant was discarded and the cells were washed and incubated with 1× Click reaction solution at room temperature for 30 min in the dark. After removal of the reaction solution, cells were incubated with 1 × Hoechst 33242 at room temperature for 10 min in the dark. Images were taken using an ortho-fluorescence microscope (BX53+DP80; Olympus, Tokyo, Japan); the proliferating cells exhibited red fluorescence, whereas their nuclei exhibited blue fluorescence.

### 4.10. Immunofluorescence 

HaCaT cells were incubated in a 24-well plate, treated according to their group, and fixed with 4% paraformaldehyde for 15 min. Cells were permeabilized with 0.2% Triton X-100 for 10 min and blocked with 10% goat serum (Solarbio, Beijing, China) for 1 h. Then, cells were incubated with antibodies against cleaved caspase-9 (AF5244, 1:200; Affinity Biosciences, Cincinnati, OH, USA) and cleaved caspase-3 (9661S, 1:100; CST, Danvers, MA, USA) overnight at 4 °C, followed by incubation with Alexa Fluor 488-conjugated Goat Anti-Rabbit IgG (H+L) (ab150077, 1:1000; Abcam, Cambridge, MA, USA) for 1 h at room temperature. Nuclei were stained with 10 μg/mL 4′, 6-diamidino-2-phenylindole (Sorlabio, Beijing, China). Cells were washed thrice with PBS, cell-climbing slices were removed, and loaded onto microscope slides, and cell images were observed using an ortho-fluorescence microscope (BX53+DP80; Olympus, Tokyo, Japan).

### 4.11. Western Blotting Analysis

After removing the supernatant and washing, HaCaT cells were lysed with the RIPA lysis buffer supplemented with 1% phenylmethylsulfonyl fluoride and phosphatase inhibitor for 30 min on ice. Cells were collected in centrifuge tubes using a cell scraper and centrifuged at 12,000× *g* for 10 min at 4 °C to obtain the total protein extract. Proteins were quantified using the BCA kit (Generay Biotechnology Co., Ltd, Shanghai, China). Protein samples were transferred to a polyvinylidene difluoride membrane (Merck Millipore, Burlington, MA, USA) using the semi-dry method after polyacrylamide gel electrophoresis. A protein-free rapid blocking buffer (EpiZyme, Shanghai, China) was used to block the membrane for 15 min and incubated overnight with the primary antibodies, p38 (sc-7972, 1:200; Santa Cruz, CA, USA), p-p38 (sc-7973, 1:200; Santa Cruz, CA, USA), JNK (sc-7345, 1:200; Santa Cruz, CA, USA), p-JNK (sc-6254, 1:200; Santa Cruz, CA, USA), ERK (sc-514302, 1:200; Santa Cruz, CA, USA), p-ERK (sc-136521, 1:200; Santa Cruz, CA, USA), p53 (2524S, 1:1000; CST, MA, USA), GAPDH (sc-365062, 1:200; Santa Cruz, CA, USA), and β-tubulin (10094-1-AP, 1:3000; Proteinech, Wuhan, China), at 4 °C, followed by incubation with secondary antibodies at 4 °C for 30 min. Protein bands were visualized using enhanced chemiluminescence Western blotting substrate reagent (7 Sea Biotech, Shanghai, China), according to the manufacturer’s instructions. Protein bands were visualized using a ChemiDoc MP Imaging System (Bio-Rad, Richmond, CA, USA).

### 4.12. Statistical Analysis

Data are represented as the mean ± standard deviation. All experiments had a minimum of three replicates. Data were analyzed using IBM SPSS Statistics (version 26.0), and one-way analysis of variance with Bonferroni post-hoc test was used to compare the groups. Statistical significance was set at *p* < 0.05.

## 5. Conclusions

In conclusion, we found that DNPs ameliorated UVB-induced acute photodamage in HaCaT cells. Moreover, DNPs inhibited UVB-induced oxidative stress, cell cycle arrest, and apoptosis by decreasing ROS production and inhibiting *MAPK* pathway activation and *p53* expression. They also enhanced the antioxidant enzyme activity and prevented the impairment of the viability and proliferation of HaCaT cells. Therefore, DNPs can be potential candidates for the treatment of UVB-induced photodamage.

## Data Availability

All relevant data are within the manuscript.
